# Assessing resource use: a case study with the Human Disease Ontology

**DOI:** 10.1093/database/baad007

**Published:** 2023-02-28

**Authors:** J. Allen Baron, Lynn M Schriml

**Affiliations:** University of Maryland School of Medicine, Institute for Genome Sciences, 670 W. Baltimore St., HSFIII, Baltimore, MD 21201, USA; University of Maryland School of Medicine, Institute for Genome Sciences, 670 W. Baltimore St., HSFIII, Baltimore, MD 21201, USA

## Abstract

As a genomic resource provider, grappling with getting a handle on how your resource is utilized can be extremely challenging. At the same time, being able to thus document the plethora of use cases is vital to demonstrate sustainability. Herein, we describe a flexible workflow, built on readily available software, that the Human Disease Ontology (DO) project has utilized to transition to semi-automated methods to identify uses of the ontology in the published literature. The novel R package DO.utils (https://github.com/DiseaseOntology/DO.utils) has been devised with a small set of key functions to support our usage workflow in combination with Google Sheets. Use of this workflow has resulted in a 3-fold increase in the number of identified publications that use the DO and has provided novel usage insights that offer new research directions and reveal a clearer picture of the DO’s use and scientific impact. The DO’s resource use assessment workflow and the supporting software are designed to be useful to other resources, including databases, software tools, method providers and other web resources, to achieve similar results.

**Database URL**: https://github.com/DiseaseOntology/DO.utils

## Introduction

Biomedical resources including databases, knowledge bases, software tools, method providers, ontologies and other web resources continue to proliferate, as do the communities they serve. Tracking resource usage metrics is key to identify areas of growth, to substantiate utility and to inform future development. Identifying how, by whom and for what purposes a resource is utilized has become a significant challenge across the biomedical domain. Certainly, there is not one single approach to determine usage, nor is it feasible for a biomedical resource to manually maintain an up-to-date and complete-as-possible list of uses to understand usage and assess the scientific impact of a resource.

The Human Disease Ontology (DO, https://disease-ontology.org/) (1), established in 2003, is a National Human Genome Research Institutefunded genomic resource and knowledge base that provides the biomedical community with a comprehensive, expertly curated, computationally tractable disease knowledge base. The DO semantically classifies the breadth of human diseases (11 181 diseases—December 2022 release) and integrates disease and clinical vocabularies through extensive cross mapping of DO terms to Medical Subject Headings (MeSH), International Classification of Diseases (ICD)-Ninth Revision (ICD-9), ICD-10, National Cancer Institute (NCI) Thesaurus, Systematized Nomenclature of Medicine Clinical Terms (SNOMED CT), Online Mendelian Inheritance in Man (OMIM), Genetic and Rare Diseases (GARD), Orphanet and ICD for Oncology (ICD-O) clinical vocabulary terms. The DO provides a stable framework for the advanced analysis of disease through curated and automated import of non-disease ontologies using ROBOT, a tool for automating ontology workflows, to define logical axioms describing anatomical, genetic, clinical and environmental factors associated with disease (2). Expansion of the DO from a small research project into a community-driven genomic resource and knowledge base, with the DO’s initial National Institutes of Health (NIH) funding, necessitated establishment of a formal process to track the DO’s expanding user base. In 2008, we established a publication collection (https://www.ncbi.nlm.nih.gov/myncbi/browse/collection/49204559/) at MyNCBI, a National Center for Biotechnology Information (NCBI) tool that enables automated, customizable searches of NCBI databases and tracking of one’s publications (https://www.ncbi.nlm.nih.gov/myncbi/), and began manually adding publications from PubMed that cited one of the DO project publications, along with publications identified in a weekly automated PubMed search (‘Disease Ontology’ not Infectious Disease Ontology (IDO); that were manually confirmed to use the DO. We also began to publish on the DO website citation statistics (https://disease-ontology.org/about/statistics and https://disease-ontology.org/community/publications) and lists of collaborators (https://disease-ontology.org/community/collaborators) and use cases (https://disease-ontology.org/community/use-cases) identified during manual review. By 2021, this approach had identified 790 publications that either cited a DO publication and/or used the DO project, including 278 ontologies and other biomedical resources that actively utilized the DO.

**Figure 1.**
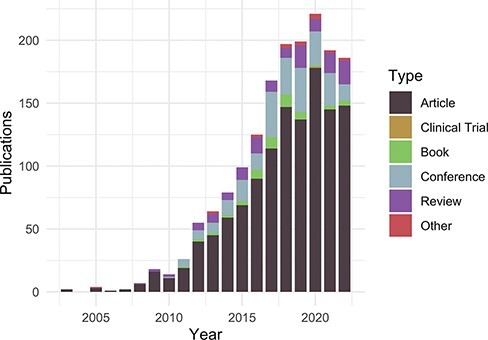
Publications citing the DO each year from PubMed and Scopus organized by publication type (https://disease-ontology.org/about/pubs_by_year). ‘Other’ publications include a small number of preprints indexed in PubMed and Scopus. The slight downward trend from 2020 to 2022 reflects a spike in publications due to COVID (2020) and incomplete publication information (2022). In November 2022, there were 1659 total unique citations to one or more of DO’s official publications.

**Figure 2.**
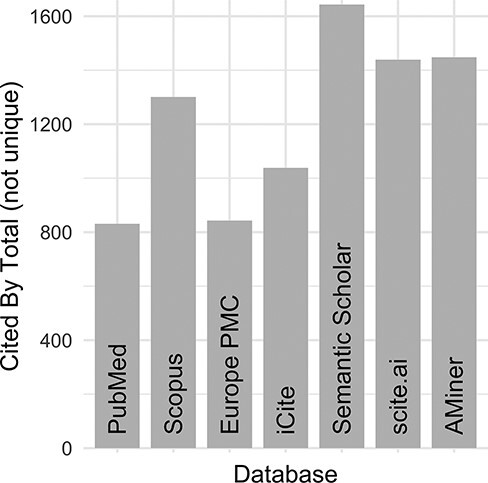
DO official publication ‘cited by’ counts. The total number of citations to each of the DO official publications published by 2021 (total = 8, https://disease-ontology.org/community/publications) were manually obtained online from each provider in August 2021. Publications from Web of Science were excluded because the University of Maryland School of Medicine does not have institutional access.

Despite these initial efforts to maintain an ‘up-to-date and complete-as-possible list of uses’, with subsequent NIH funding (https://disease-ontology.org/about/) and substantial growth of the DO’s citations (Figure 1), collaborators and use cases, it became increasingly difficult, time-intensive and, indeed, impossible to manually curate the breadth of DO uses. To address the challenge of use tracking at scale, we recently sought a new approach that would reduce manual curation effort, would not require specialized software, would require minimal time spent programming, would build and expand on the in-depth information derived from previous efforts and could be easily generalized for use by other biomedical resources. A major hurdle in the quest for an ‘accurate literature-based impact measure’ is that reporting of citations of one’s work, or coverage (3, 4), varies across biomedical literature databases, as noted in the differences in the number of citations reported in PubMed, Scopus and Google Scholar. Examination of total citation counts for all eight of the DO project publications published at that time (2021) across the biomedical literature databases [PubMed (5), Scopus (https://www.scopus.com/), Europe PMC (6), iCite (7), Semantic Scholar (8), scite.ai (https://scite.ai/) and AMiner (https://www.aminer.org/); Figure 2] revealed the need for this approach to expand beyond PubMed ‘cited by’ lists and manual text search capabilities, as these were not capturing the full breadth of uses for the project.

Here, we describe a simple, open-source, semi-automated workflow that captures a more comprehensive representation of published uses and facilitates faster curation built on a small set of key functions in the novel R package DO.utils and Google Sheets. We have utilized this approach to gain a more informed understanding of the breadth and depth of DO’s uses and DO’s impact on scientific research. This approach provides novel insights—identifying areas of focus for future work and potential collaborators who might assist in improving the DO. Our intent is to offer this workflow as an approach to be used by other biomedical resources to better understand how their resources are used, to gain insights on user communities and to demonstrate scientific impact.

## A simple workflow to assess use

In its most basic form, this use assessment workflow has three main steps: (i) obtaining records of research that have cited and/or used the biomedical resource; (ii) curating those records to verify and capture details of use and (iii) evaluating the nature and trends of use from the captured and curated information. This workflow is supported by automation developed as part of a growing R package named DO.utils (https://github.com/DiseaseOntology/DO.utils) using the existing code from other R packages as much as possible and by utilizing functions available in Google Sheets. DO.utils was designed to assist in updating, maintaining and analyzing the DO, a broader purpose than that covered by this workflow. Nevertheless, the code in DO.utils that supports this use assessment workflow is of a more general design and can readily be employed by resources beyond the DO and its users. However, DO.utils is not intended to be a fully automated scientometrics or bibliometrics package, and some decisions and curation are expected as part of this workflow. DO.utils will continue to be updated and expanded on a quarterly cycle as needed to better support this workflow. For the software set-up needed to execute this workflow, refer to the ‘Software Set-up’ section at the end of this publication.

### Obtaining use records

The first step in the resource use assessment workflow is to obtain records of research that cited and/or used the resource from published literature databases, arguably ‘the coin of the academic realm’ (9), and to prepare the records for efficient curation.

DO.utils provides three mechanisms to identify publications that have cited and/or used a resource: (i) automated retrieval of publications that cite a publication produced by the biomedical resource, referred to hereafter as ‘cited by’ publications, (ii) automated publication retrieval based on a keyword literature search and (iii) inclusion of a manually curated MyNCBI collection.

Automated retrieval of ‘cited by’ records for one or more publications produced by the biomedical resource is straightforward. For ‘cited by’ records from PubMed, provide the PubMed identifiers (PMIDs) of the resource’s published works to DO.utils’ citedby_pubmed(). For ‘cited by’ records from Scopus, use the similarly named function citedby_scopus() with the titles of a resource’s published works as input. These functions use the rentrez (10) and rscopus (https://CRAN.R-project.org/package=rscopus) R packages under the hood and return records as complex list structures that can be formatted to tables with as_tibble(), retaining all information but in somewhat complex nested structures, or with tidy_pub_records() for neater, but more limited information. If more than one resource publication PMID or title is used as input, both citedby_*() functions will optionally identify the publication that is ‘cited by’ each record in a ‘cites’ column.

Automated retrieval of publications that may have used a biomedical resource can also be achieved with a keyword literature search against the PubMed, PubMed Central or Europe PMC databases using search_pubmed() and search_pmc() from DO.utils or epmc_search() from the europepmc R package (https://CRAN.R-project.org/package=europepmc), respectively. These functions accept the same search parameters as the databases they access. epmc_search() returns detailed publication records in a tabular format, while search_pubmed() and search_pmc() provide only PMIDs of the publications. Full publication records can be obtained by providing the PMIDs to the pubmed_search() of DO.utils, which returns data in the same format as citedby_pubmed() and can be formatted as a table in the same way.

A MyNCBI collection can be utilized to manually build a collection of PubMed publications that cite and/or mention the usage of a resource within their publication. Creating and adding to a MyNCBI collection is outlined in the ‘Collections’ chapter in the My NCBI Help book (https://www.ncbi.nlm.nih.gov/books/NBK53590/). When ‘cited by’ and search approaches are utilized, a MyNCBI collection may be of limited value unless additional records are identified outside of these approaches. The decision to include MyNCBI collections in this workflow is primarily to take advantage of manually identified records curated by the DO team prior to 2021. To import a MyNCBI collection into R and format it for curation, first manually download the collection. Open the collection in a web browser and select ‘Send to:’, and then, in the ‘File’ option, choose ‘Summary (text)’ as the format and click ‘Create File’. Parse the records in the file into R by providing the file path of the downloaded file to the read_pubmed_txt() function of DO.utils.

If only one of the approaches described to identify publications using a resource is utilized, the resulting records can be saved to a Google Sheet (or another file format) for review as described in the ‘Curating Use Information’ section. However, when multiple approaches are used, the records must be deduplicated or merged. This requires comparing the records to one another, which can be challenging because different publications may have the same title (e.g. a conference report and a later published article), mismatched titles or variable other publication details such as the journal name, volume and page numbers. To avoid these matching difficulties, records can be compared using match_citations() from DO.utils. This function takes two sets of publication records, identifies matches using standard record identifiers found in the data (e.g. PMIDs and DOIs) and returns the numeric position of the records in the second record that matches the first record (similar to match() from base R). Since records may have more than one identifier, the matching algorithm compares available identifiers in a prioritized manner as follows: PubMed IDs > PubMed Central IDs > DOIs > Scopus EIDs. DO.utils provides collapse_col() for limited merging and deduplication of matched records, which should then be saved for subsequent curation. For reasons described in the ‘Curating Use Information’ section, saving to Google Sheets is recommended for curation. From R, this can be accomplished with the googlesheets4 R package (https://CRAN.R-project.org/package=googlesheets4). Examples of how to accomplish the tasks of this section can be found in the ‘Assessing Resource Use: Obtaining Use Records’ tutorial accompanying DO.utils’ documentation (https://diseaseontology.github.io/DO.utils/articles/obtain_use_records.html) or in the DO case study code, available on GitHub at https://github.com/DiseaseOntology/assessing_DO_use and the persistent open-access repository Zenodo (https://doi.org/10.5281/zenodo.7467640).

### Curating use information

The second step in this workflow focuses on curating use information. This is best accomplished by reviewing the full text of published works obtained in the previous step. Minimally, manual expert curation should verify that a publication actually used the resource. Google Sheets provides a number of advantages that make curation simple, fast and flexible while minimizing data entry errors that might hinder later evaluation. First, identifiers can be converted to clickable links, making access to full-text publications faster. This can be done before saving the file with the DO.utils’ build_hyperlink() function. Second, data validation and Google Sheets’ standard functions can be used to enhance both data standardization and curation speed in columns either manually added after saving the data or added as empty placeholder columns before saving the data by providing column names to append_empty_col(). When appending to existing sheets, provide all column names from the sheet and the tabular data to be appended to append_empty_col(), in order to avoid data saving problems. The columns to add for curation will depend on the information each resource desires to collect. Examples are provided in the case study with DO where each column is designed to answer a specific question. To add data validation to a column, simply select the column and then ‘Data Validation’ from the ‘Data’ menu. Then, set up the valid values using one of the implementations described later. Entry into validated columns can be done using the dropdown menu in each cell, or by typing in the cell, partial text matches will be suggested. Partial text matching can be particularly useful when a long list of values are valid (such as when using an ontology).

Validation Implementation #1 strictly ensures that only controlled values can be entered. To set up this implementation, choose the ‘On invalid data: Reject input’ setting and either specify values as a list or create a separate column with accepted values and add that range in the ‘List from a range’ box. This implementation could optionally be made less strict with the ‘On invalid data: Show warning’ setting, but it is not recommended. If new values are desired, add them to the list or column.

Validation Implementation #2 provides dynamically updated data suggestions extracted directly from data entered into the curation column and recognizes only one input per cell. This can be set up by using the ‘On invalid data: Show warning’ setting on the curation column and adding the function =sort(unique(<curation range>)) to the top cell of a designated validation column, replacing <curation range> with the full range of the curation column minus the heading. The dynamic update of ‘valid’ data values directly from data entered into the curation column can be very useful when valid values are unknown and many new values are likely to be added, but this will reduce data standardization. Consider using this implementation during early curation efforts, later switching to Implementation #1 for more robust standardization.

Validation Implementation #3 provides the dynamically updated data suggestions of Implementation #2 while also allowing multiple inputs in a single cell. Set-up is the same as set-up for Implementation #2 except the function in the top cell of the validation range should be =sort(unique(transpose(arrayformula(trim(split(join(" | ",< curation range>)," | ")))))), and multiple curation inputs to a single cell should be separated by a pipe delimiter ("|"). Note that space around the delimiter can be added for readability and is removed by this validation function. Other delimiters can also be used by replacing the pipe in the validation function. There is no way to convert this implementation to Implementation #1 and use a more controlled vocabulary. Furthermore, Google Sheets will only suggest matches while typing in a cell until the delimiter is typed, so a curator must either identify all desired values before typing the delimiter or use another cell to obtain additional suggestions. For Implementation #2 and #3, values incorrectly typed will become suggested options so care should be taken. In practice, we have found it easy to identify and edit incorrectly typed entries and have appreciated the significant speed boost and flexibility of these approaches. All of these implementations of data validation can also be explored in an interactive ‘Validation Sandbox’ Google Sheet (https://docs.google.com/spreadsheets/d/1WBPYdAEOYLdR_qVYw7GYHsPQxGEo58SGz0tpEtPfU2Y).

### Evaluating use

The final step in the resource use assessment workflow is to evaluate the type of usage. It is in this step that the value of the workflow can be fully observed. However, the information derived will depend on the specifics of the information curated. Some examples of assessments and how they may prove useful are listed in Table 1. The ‘Curating Information’ section under ‘Case Study: the Human Disease Ontology’ provides specific examples.

**Table 1.** Example assessments for evaluating resource use in the published literature

Assessment	Depends on	Useful for	Difficulty
Count of ‘cited by’ or use records	Obtaining records	• Demonstrating essential impact	Easy
List of research authors	Obtaining records	• Identifying user community and potential collaborators	Moderate
Curated details of uses by biomedical resources	Curating information	• Demonstrating breadth of use and scientific trust	Moderate
		• Identifying user community and potential collaborators	
Summary of uses by research area	Curating information	• Demonstrating importance in specific fields	Moderate
		• Identifying focus areas for future work	
Example use cases	Curating information	• Broadening accessibility	Moderate
		• Supporting claims and uses	

DO.utils contains two functions to aid in use evaluation:

- plot_citedby() to create a plot of ‘cited by’ records over time by type of publication.
- count_delim() to summarize data columns with multiple, delimited inputs as created using Validation Implementation #3.

## Case study: the Human Disease Ontology

The code and data input used to execute this workflow for the DO, along with resulting data and graphical outputs, are available from GitHub (https://github.com/DiseaseOntology/assessing_DO_use) or Zenodo (https://doi.org/10.5281/zenodo.7467640) and in a ‘DO_uses’ Google Sheet (https://docs.google.com/spreadsheets/d/1soEnbGY2uVVDEC_xKOpjs9WQg-wQcLiXqmh_iJ-2qsM/).

### Obtaining records

While developing this workflow, we noted that 20% of the 790 publications in the DO’s MyNCBI collection used the DO but did not appropriately cite a DO project paper. We, therefore, sought to implement all three methods—‘cited by’, literature search and incorporation of DO’s manually curated MyNCBI list—to capture as many publications as possible that used the DO and to merge these to avoid record duplication. The eight DO project papers published prior to 2022 (https://disease-ontology.org/community/publications) were used to identify 861 unique ‘cited by’ records from PubMed and 1400 from Scopus. We merged these records with the publications in DO’s curated MyNCBI collection, resulting in a substantial increase of the total unique ‘cited by’ records from 790 to >1500.

**Figure 3.**
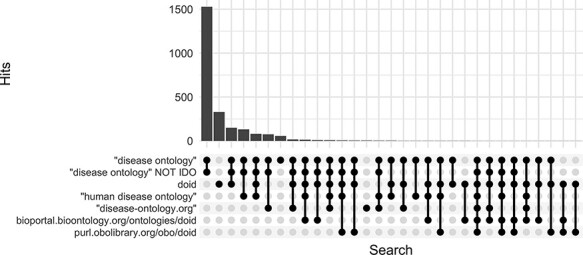
Comparison of keyword search hits at Europe PMC using the ‘europepmc’ R package (completed 9 November 2022).

**Figure 4.**
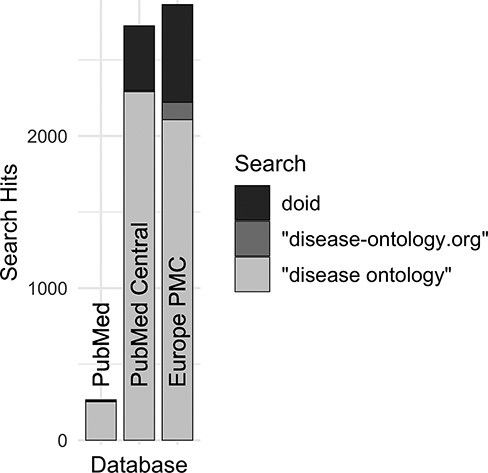
Comparison of search hits at different publication databases for the three keyword searches with the greatest number of hits at Europe PMC. These case-insensitive searches identify mentions of DO’s specific identifier, the official website or the ontology itself without ‘Human’, which is often left out by users.

Along with DO’s long established search phrase (‘Disease Ontology’ not IDO), additional sets of keyword searches were tested against Europe PMC (Figure 3) to discover which would best identify publications that mention and potentially used the DO. From these, three keyword searches were chosen because they remained specific to the DO and recovered the most unique publications: (i) DOID, the specific identifier for disease terms in the DO, (ii) ‘disease ontology’ and (iii) ‘disease-ontology.org’ (Figure 4). Using these three distinct searches, against PubMed, PubMed Central and Europe PMC has identified 2804 potential publications using the DO. Of these 1775 (51%) were new; they had not previously been included in ‘cited by’ records or the DO’s MyNCBI collection. These records have been added to the DO team’s curation queue for further review. All together records for 3432 publications that have cited and potentially used the DO were obtained.

### Curating information

In order to gain a deeper understanding of how the DO is being used, the DO team initiated a deep dive to assess the merged ‘cited by’ and MyNCBI collection records published since September 2021. To support curation, publication identifiers were converted to clickable hyperlinks and the merged publication record data were saved to a ‘DO_uses’ Google Sheet (https://docs.google.com/spreadsheets/d/1soEnbGY2uVVDEC_xKOpjs9WQg-wQcLiXqmh_iJ-2qsM/, see ‘cited_by’ sheet). Then, columns for curation were added along with data validation to address the following questions:

1. Does the publication actually use DO and to what degree? (column: uses_DO; Validation Implementation #1 with accepted values of ‘yes’, ‘minimal’, ‘mention’, ‘unclear’ and ‘no’)
2. What type of use(s) does/do this publication describe? (column: use_type; Validation Implementation #3, multi-input)
  Additional columns were added to capture details of DO use by other biomedical resources (a.k.a. ‘tools’):
    - The name of the tool (column: tool_name; no data validation).
    - The direct URL to the tool on the web or the URL to the publication if it is not available on the web, which is common for methods (column: tool_url; no data validation).
    - The role the tool plays in research (column: tool_role; Validation Implementation #3, multi-input).
    - A unique identifier assigned by the DO team (column: tool_ID; no data validation).
3. What research area(s) is/are covered by this publication? (column: research_area; Validation Implementation #3, multi-input)
4. What disease, if any, is the focus of this publication? (column: disease; Validation Implementation #1 with accepted values including all valid diseases from the 29 September 2022 DO release, https://github.com/DiseaseOntology/HumanDiseaseOntology/tree/v2022-09-29)

An additional column was added to capture general review notes (no data validation).

Validation ranges for each curation column with data validation applied were set up with matching column names in a second tab of the same Google Sheet (‘cited_by-validation’ sheet). The data validation columns with Implementation #1 had predefined acceptable values added, while those with Implementation #3 began as empty ranges containing only the corresponding function described in the ‘Curating Use Information’ section of the workflow. The accepted values, at the time of writing for each of the columns described earlier, are available in Tables 2–3 and Supplementary Tables 1 and 2. The full list of accepted values for ‘disease’ was generated by pasting all disease terms and identifiers from the 29 September 2022 DO release into the corresponding validation column (not shown), demonstrating that Google Sheets can handle large validation sets (>11 000 values).

**Table 2.** Summary of DO use in ‘cited by’ publications from September 2021 to October 2022

Review Status	Uses DO	Count	Percentage
Not yet reviewed		53	
Inaccessible		26	
Reviewed	Yes	75	60.0
	Minimal	5	4.0
	Mention	33	26.4
	Unclear	7	5.6
	No	5	4.0
	Total	125	

The DO team’s curation of usage follows a decision tree that initially involves review of the title and abstract of the publication to determine if the paper describes a biomedical resource or primary research (Question #2). Biomedical resources are frequently named in the title, and the URL for biomedical resources is often included in the abstract. Next, the text of a paper is searched for keywords, such as ‘ontology’, ‘disease ontology’ or ‘DOID’ (the specific identifier for disease terms in the DO) to determine if and how the DO was used (Question #1). If the DO is more than simply mentioned and the paper is reporting a database or other biomedical resource, additional details of the resource are captured and the resource website is examined to aid in identification of how the DO is utilized (Question #2, A–D). Finally, for all publications reviewed, one or more areas of research are recorded (Question #3). Usage, for the majority of papers we examine, is quickly ascertained from the title and abstract of papers. With clickable links and this data validation scheme promoting flexible, yet structured, data entry, the DO team has curated the usage information described for all publications over the last year while reducing the time it takes for review to 3–12 minutes per publication (mean: 7 minutes), excluding paywalled publications. For ∼200 publications in this set of papers that used the DO, this amounts to only 6 hours of curation per quarter (or half an hour per week), which we consider quite reasonable for the depth of information obtained.

### Evaluating use 

**Figure 5.**
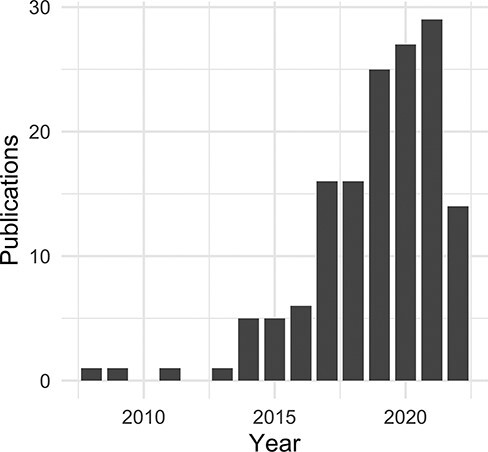
Non-coding RNA publications citing the DO by year.

For this publication, the evaluation dataset of curated, citation-based use data has been limited to the time period from September 2021 to October 2022. We identified a direct use of the DO for nearly two-thirds of the reviewed publications (Table 2). Among these, the majority describe biomedical resources (databases, software tools and other web resources) and methodologies (algorithms, frameworks, software tools, workflows and state-of-the-art methods) (https://disease-ontology.org/community/use-cases), followed closely by primary analyses (Table 3). The DO is utilized by other biomedical resources to classify and annotate disease-related data, to extract and align biomedical data across model organism databases, as a data source in natural language processing applications [machine learning (ML)], and to aid in prediction, analysis and visualization studies (Supplementary Table 1). Most publications utilize all of the diseases in the DO or search for diseases based on characteristics (Supplementary Table 3). Those citations that are disease-specific were, not surprisingly, primarily focused on cancer and coronavirus disease 2019 (COVID-19). It was noted that the DO is utilized broadly for disease–genetic association studies, drug–disease research including drug–drug interactions and non-coding RNA (ncRNA) databases (Supplementary Table 2). Further review of ‘RNA’ associated publications revealed a noticeable increase of ncRNA databases, particularly in the last 3 years (Figure 5 and Supplementary Figure 1).

**Table 3.** Summary of the types of biomedical resources or research created with use of the DO in ‘cited by’ publications from September 2021 to October 2022

Use type	Count	Percentage
Resources^a^		
Corpus	1	1.1
Database	22	23.2
Knowledge base	5	5.3
Method	3	3.2
Method > algorithm	3	3.2
Method > ML	7	7.4
Method > ML > neural network	2	2.1
Online platform	17	17.9
Ontology	2	2.1
Software	6	6.3
Software > R package	1	1.1
Web app	6	6.3
Other		
Dataset	1	1.1
Primary analysis	16	16.8
Meta-analysis	3	3.2
Total	95	

a More than one resource may be described in a publication as part of a project (e.g. a database and an online platform). These resources were generated by 43 unique projects.

## Software set-up

DO.utils’ software dependencies include R and a number of R-based packages that install automatically with DO.utils. Detailed instructions are available in a DO.utils’ tutorial at https://diseaseontology.github.io/DO.utils/articles/obtain_use_records.html. To set up DO.utils for use, first download and install R from The Comprehensive R Archive Network (CRAN; https://cran.r-project.org/). Installation of an integrated development environment, such as RStudio (https://posit.co/products/open-source/rstudio/), is recommended but optional. Next, install the devtools R package by running install.packages(‘devtools’) in R/RStudio. DO.utils can then be installed from GitHub with devtools::install_github(‘DiseaseOntology/DO.utils’). To ensure persistent access, DO.utils has also been added to the open-access repository Zenodo where specific versions can be downloaded (https://doi.org/10.5281/zenodo.7467668) and then installed with devtools::install_git(<path>), replacing <path> with the local path to the DO.utils download. Dependencies should install automatically.

DO.utils uses two Application Programming Interfaces (APIs) to obtain records: the Entrez Utilities API of NCBI and the Scopus search API. Access to NCBI’s Entrez Utilities API is free and accessible to all without any additional set-up. However, NCBI highly recommends that users obtain a free API key to avoid rate limit issues. Details are described in NCBI’s online ‘Entrez Programming Utilities Help’ book (https://www.ncbi.nlm.nih.gov/books/NBK25497/). Scopus is a proprietary service that requires an institutional subscription and two API keys, one that is freely available to all subscribers (https://dev.elsevier.com/) and a second ‘institutional token’ (insttoken), which must be requested from Scopus by an institution’s librarian or information officer (https://dev.elsevier.com/support.html). Without the insttoken, citation records will not be accessible and only ‘cited by’ counts can be obtained using the rscopus package.

## Discussion

Illuminating the diversity of DO uses and transforming a once manually intensive activity through this new workflow have greatly increased the project’s ability to capture and examine trends in usage. Augmenting the DO’s production pipeline, as outlined, now provides a semi-automated, informed approach for tracking DO uses that can be utilized by other biomedical resources. Tracking shifts in usage over time, through this approach, informs areas of expanded utility and guides future development of the DO, such as the planned expansion of the DO’s representation of diseases associated with ncRNAs in 2023. Examination of DO uses has revealed over 50 biomedical ontologies using the DO, by either reusing DO’s terms and/or IDs, using unique URL-based IDs, e.g. http://purl.obolibrary.org/obo/DOID_, or mapping to DOIDs as ontology cross-references (xrefs), synonyms or annotations. Identification of software developed using the DO has further expanded opportunities for integrating ML and artificial intelligence software to aid in the DO’s production pipeline through new collaborative efforts.

## Future work

In the future, we plan to develop a second R package that focuses solely on assessing biomedical resource use to augment the functions of DO.utils. We will also expand upon the automation in this workflow as time permits. New functions we plan to integrate will support formatting and merging citations and will simplify the API key management and saving of data. Future planned functions will enable analyzing curated and other citation information, centralizing search functions to execute multiple searches across multiple databases while returning compiled results and expanding ‘cited by’ and search to more databases. The DO’s ‘cited by’ results will be further expanded through the incorporation of results from lens.org and/or Semantic Scholar. Search results will also be included in the DO team’s quarterly use assessment curated reviews.

## Supplementary Material

baad007_Supp

## Data Availability

The DO.utils R package that supports the automated steps of this workflow can be found on GitHub (https://github.com/DiseaseOntology/DO.utils) or on the persistent, open-access repository Zenodo (https://doi.org/10.5281/zenodo.7467668). Detailed documentation is also available on the web at https://diseaseontology.github.io/DO.utils/, including a step-by-step tutorial (https://diseaseontology.github.io/DO.utils/articles/obtain_use_records.html). The code, data and graphics of the DO case study can be found on GitHub at https://github.com/DiseaseOntology/assessing_DO_use or on Zenodo (https://doi.org/10.5281/zenodo.7467640). All code, data and graphics are available under the Creative Commons CC0 1.0 Universal license (https://github.com/DiseaseOntology/HumanDiseaseOntology/blob/main/LICENSE). The Google Sheet used in the DO case study for curation and analysis is available at https://docs.google.com/spreadsheets/d/1soEnbGY2uVVDEC_xKOpjs9WQg-wQcLiXqmh_iJ-2qsM/, and the Validation Sandbox Google Sheet for exploring different data validation implementations in Google Sheets is available at https://docs.google.com/spreadsheets/d/1WBPYdAEOYLdR_qVYw7GYHsPQxGEo58SGz0tpEtPfU2Y/.

## References

[1] Schriml L.M. , Munro J.B., Schor M. et al. (2022) The Human Disease Ontology 2022 update. *Nucleic Acids Res.*, 50, D1255–D1261. 34755882 10.1093/nar/gkab1063 PMC8728220

[2] Jackson R.C. , Balhoff J.P., Douglass E. et al. (2019) ROBOT: a tool for automating ontology workflows. *BMC Bioinform.*, 20, 407. 10.1186/s12859-019-3002-3 PMC6664714 31357927

[3] Kokol P. and Vošner H.B. (2018) Discrepancies among Scopus, Web of Science, and PubMed coverage of funding information in medical journal articles. *J. Med. Libr. Assoc.*, 106, 81–86. 29339937 10.5195/jmla.2018.181 PMC5764597

[4] Bakkalbasi N. , Bauer K., Glover J. et al. (2006) Three options for citation tracking: Google Scholar, Scopus and Web of Science. *Biomed. Digit. Libr.*, 3, 7. 10.1186/1742-5581-3-7 PMC1533854 16805916

[5] Sayers E.W. , Bolton E.E., Brister J.R. et al. (2023) Database resources of the National Center for Biotechnology Information in 2023. *Nucleic Acids Res.* 51, D29–D38. 36370100 10.1093/nar/gkac1032 PMC9825438

[6] Ferguson C. , Araújo D., Faulk L. et al. (2021) Europe PMC in 2020. *Nucleic Acids Res.*, 49, D1507–D1514. 33180112 10.1093/nar/gkaa994 PMC7778976

[7] Hutchins B.I. , Baker K.L., Davis M.T. et al. (2019) The NIH Open Citation Collection: a public access, broad coverage resource. *PLoS Biol.*, 17, e3000385. 10.1371/journal.pbio.3000385 PMC6786512 31600197

[8] Lo K. , Wang L.L., Neumann M. et al. (2020) S2ORC: The Semantic Scholar Open Research Corpus. In: Proceedings of the 58th Annual Meeting of the Association for Computational Linguistics. Association for Computational Linguistics, Online, pp. 4969–4983.

[9] Kratz J.E. and Strasser C. (2015) Making data count. *Sci. Data*, 2, 150039. 10.1038/sdata.2015.39 PMC4550906 26396741

[10] Winter D.J. (2017) rentrez: an R package for the NCBI eUtils API. *R J.*, 9, 520–526.

