# Supplementary figures and images for: Assessing resource use: a case study with the Human Disease Ontology
Source: Database (Oxford). 2023 Feb 28;2023:baad007. doi: 10.1093/database/baad007 (PMC9972798; doi:10.1093/database/baad007)

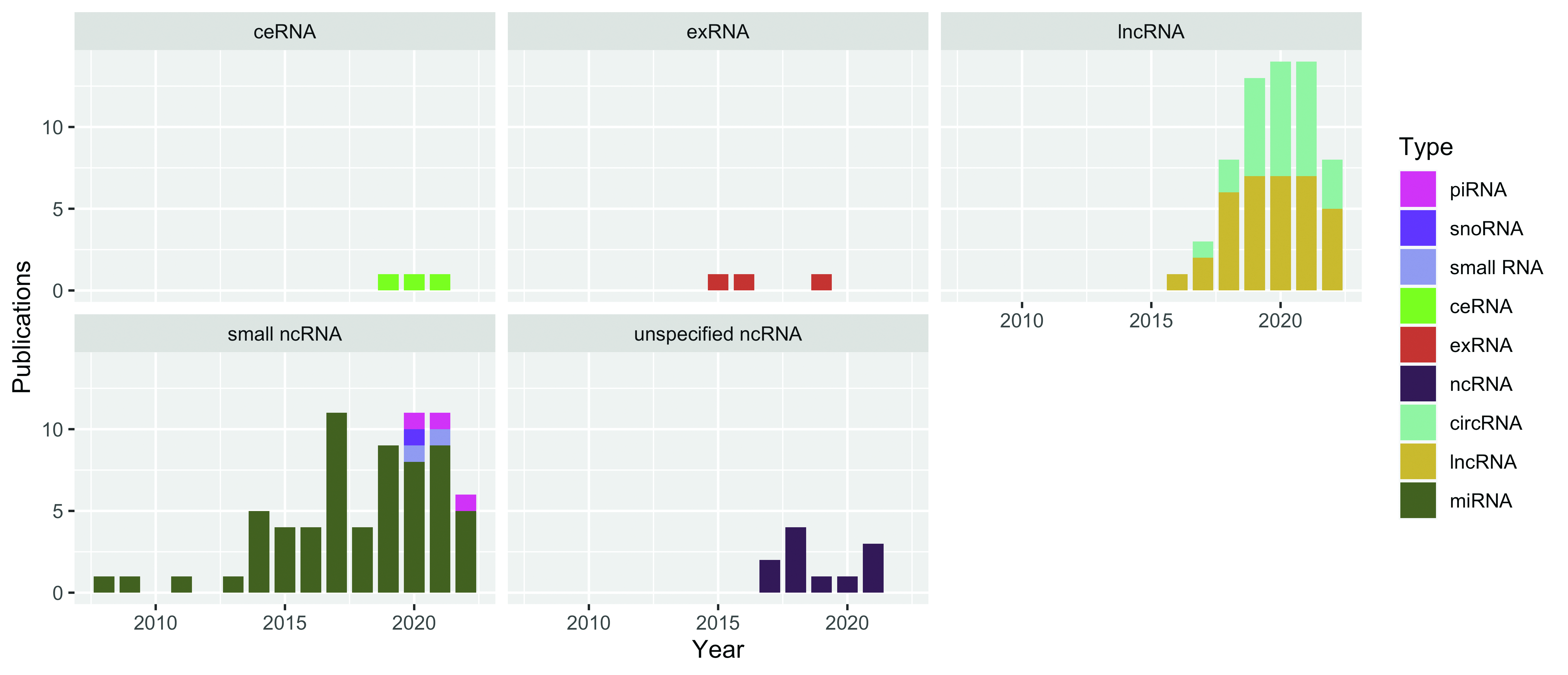

Supplement: baad007_Supp [file baad007_supp.zip › suppl_data/Supp_Figure1.tif]
